# Real-World Experience With the Angio-Seal Closure Device: Insights From Manufacturer and User Facility Device Experience Database

**DOI:** 10.1177/15266028231219226

**Published:** 2023-12-18

**Authors:** Aida Ahrari, Gerard M. Healy, Adam Min, Fahd Alkhalifah, George Oreopoulos, Kong Teng Tan, Arash Jaberi, Dheeraj K. Rajan, Sebastian Mafeld

**Affiliations:** 1 Department of Radiology, University of Toronto, Toronto, ON, Canada; 2 Joint Department of Medical Imaging, University Health Network and Sinai Health System, Toronto, ON, Canada; 3 Department of Radiology, St Vincent’s University Hospital, Dublin, Ireland; 4 Division of Vascular Surgery, University Health Network, Toronto, ON, Canada

**Keywords:** Angio-Seal, MAUDE, vascular closure devices, safety, adverse event

## Abstract

**Purpose:**

Angio-Seal (Terumo Medical Corporations, Somerset, New Jersey) device is indicated for femoral arteriotomy closure. Real-world published data on complications are limited. We present 1 year of safety events involving Angio-Seal from the US Food and Drug Administration’s post-market surveillance database of Manufacturer and User Facility Device Experience (MAUDE). Steps for managing frequent device-related problems are discussed.

**Materials and Methods:**

Angio-Seal MAUDE data from November 2019 to December 2020 was classified according to (1) mode of device failure, (2) complication, (3) treatment, and (4) Cardiovascular and Interventional Radiological Society of Europe (CIRSE) adverse event classification system.

**Results:**

There were 715 safety events, involving Angio-Seal VIP (93.1%), Evolution (5.7%), STS Plus (1.1%), and sizes 6F (62.5%) and 8F (37.5%). Failure mode involved unrecognized use of a damaged device (43.4%), failed deployment (20.1%), failed arterial advancement (6.3%), detachment of device component (4.9%), failed retraction (3.6%), operator error (1.1%), and indeterminate (20.6%). Of total, 44.8% of events were associated with patient harm. Complications involved minor blood loss (34.1%), hematoma (5.6%), significant blood loss (1.4%), and pseudoaneurysm (1.4%). Of total, 43.3% of cases required manual compression (MC), whereas 8.8% required more advanced intervention. Interventions included surgical repair (49.2%), thrombin injection (9.5%), balloon tamponade (6.3%), covered stent (4.8%), and unspecified (30.2%). Majority of safety events were CIRSE grade 1 (92.0%), followed by grades 2 (3.1%), 3 (4.6%), and 6 (deaths, 0.3%). Minority of devices were returned for manufacturer analysis (27.8%).

**Conclusions:**

The majority of safety events were associated with minor blood loss or local hematoma and could be addressed with MC alone. Most events were attributed to damaged device; however, very few devices were returned to manufacturer for analysis. This should be encouraged to allow for root cause analysis in order to improve safety profile of devices. System-level strategies for addressing barriers to under-reporting of safety events may also be considered.

**Clinical Impact:**

Our study highlights important safety events encountered in real-world practice with Angio-Seal closure device. The MAUDE database captures real-world device malfunctions not typically appreciated in conventional clinical trials. Our study provides valuable insight for clinician-users on anticipating and managing the most common device malfunctions. Additionally, our data provide feedback for manufactures to optimize product design and direct manufacturer user training to improve safety. Finally, we hope that the study promotes system-level strategies that foster reporting of safety events and undertaking of root cause analysis.

## Introduction

More than 7 million percutaneous arterial procedures are performed annually worldwide, and the common femoral artery is the most frequent access site.^1^ Although manual compression (MC) remains the gold-standard for achieving homeostasis, it may at time be time-consuming, personnel intensive, require prolonged bed rest, and less comfortable for patients.^1^ Moreover, MC may not achieve adequate hemostasis in patient demographics with obesity, anticoagulation, or antiplatelet use.^1^

Vascular closure devices (VCDs) were first introduced in the early 1990s. Vascular closure devices have the advantage of minimizing time to hemostasis, resource utilization (alleviates staff of providing MC), bed rest, as well as time to ambulation and discharge.^1^ The disadvantages include risk of device malfunction, groin infection, limb ischemia, localized thrombosis or distal embolization, as well as device cost and inherent learning curve for operation. Multiple factors influence outcomes of VCDs, including patient characteristics (obesity, anticoagulation status), intervention modality, device features, operator experience, and anatomic challenges (vessel size, degree of calcification).^1^ With the rapid expansion of VCDs, the estimate market size has surpassed 1 billion dollars in 2021.^2^ As vascular percutaneous interventions grow, VCDs continue to become increasingly more valuable.

**Figure 1.**
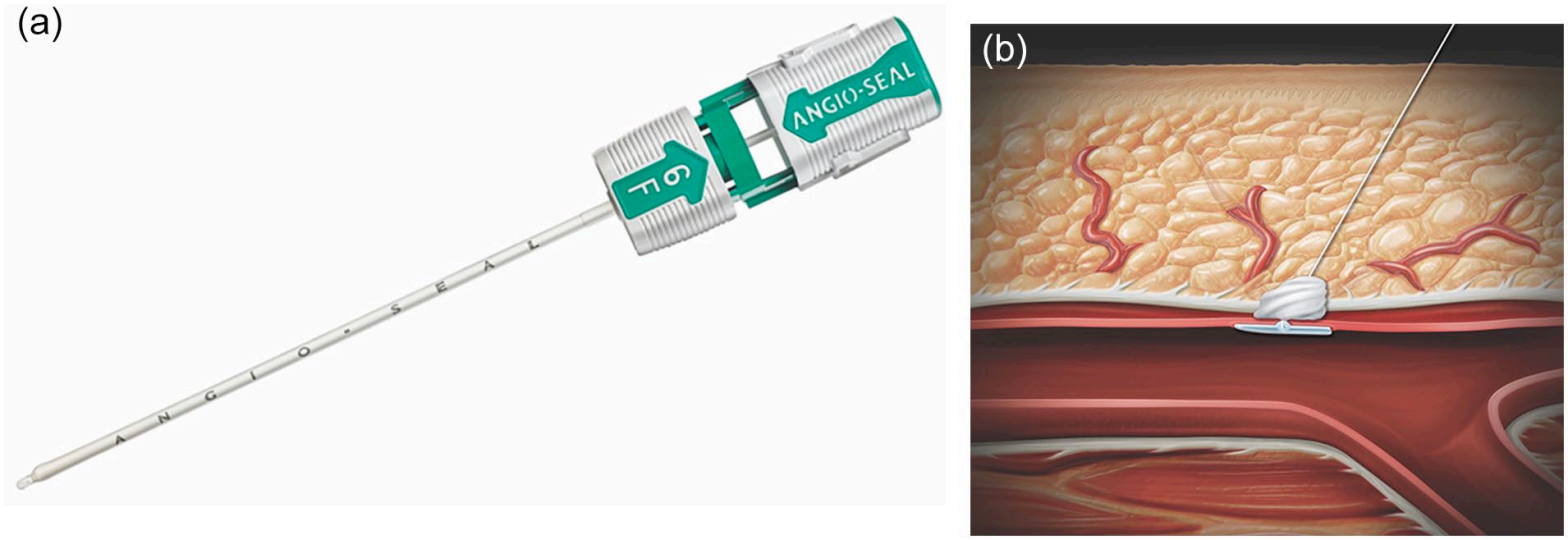
(A) Angio-Seal VIP 6F. (B) Graphic demonstrating mechanism of hemostasis. Source: Images courtesy of Terumois.com.

Angio-Seal (Terumo Interventional Systems, Somerset, New Jersey) is a VCD indicated for diagnostic and therapeutic interventions. There are 3 versions of the device: STS plus, VIP, and Evolution: introduced in 1999, 2005 and 2012, respectively. STS plus is a suture-mediated closure system, whereas VIP and Evolution utilize a bioabsorbable closure mechanism.^1^ Evolution is the newest version with a larger plug for faster closure time and a more user-friendly design.^1^ Greater than 75% of Angio-Seal devices used worldwide are VIP.^1^ The Angio-Seal VIP and Evolution systems are composed of the following components: an insertion sheath, arteriotomy locator (modified dilator), guidewire, absorbable collagen sponge, and absorbable polymer anchor connected by an absorbable suture (Figure 1). The device operates as a passive approximator that deploys a collagen plug over the arteriotomy site with the aid of an intraluminal anchor.^1^ After intraluminal placement is confirmed, an anchor abuts the wall and a collagen plug is deployed over the arteriotomy. The plug expands when it enters the subcutaneous tissue and accelerates the clotting cascade. The anchor and plug are resorbed over time. Angio-Seal has been approved to close arteriotomy sites up to 8F; although in practice, it is used to close larger-caliber arteriotomies ranging from 9 to 12F with good efficacy.^3^

Clinical trials of Angio-Seal suggest low complication rates.^4–9^ Yet, real-world experience may reveal new safety issues not previously appreciated in clinical trials.^10^ Over the last decade, numerous devices have been recalled following US Food and Drug Administration (FDA) approval due to safety concerns identified in clinical practice.^11,12^ This highlights the need for post-market surveillance, which may reveal issues not typically assessed for in clinical trials. Herein, we present real-world safety events associated with Angio-Seal using the FDA’s Manufacturer and User Facility Device Experience (MAUDE) database.^13^ We also propose strategies to mitigate and manage complications associated with Angio-Seal use.

## Materials and Methods

### MAUDE Database

The MAUDE database stores post-market adverse events involving medical devices.^13,14^ The database is updated monthly by reports from manufacturers, device users, health care professionals, and patients. Reports contain information about the device and narrative description of the safety event. The database is reviewed routinely by the FDA and manufacturers to address device-related safety events. The FDA issues safety alerts or recalls if a device is found to be defective. Although the database does not have utility in establishing definitive adverse event rates as it relies on clinician reporting, it can provide insight into the mechanism of device associated failure. There are a number of studies that have explored the MAUDE database for obtaining real-world safety data on various devices.^15–22^

### Data Collection

The MAUDE database was searched on February 2, 2022 using search terms “Angio-Seal,” “Angioseal,” and “Angio Seal.” Data from November 2019 to December 2020 were independently evaluated by 2 radiology resident reviewers. After excluding duplicate (68) and incomplete entries (82), 715 reports were included in the final analysis. Reports were classified according to (1) mode of device failure, (2) type of complication, (3) treatment modality used to manage the complication, and (4) level of associated patient impact using the Society of Interventional Radiology (SIR) and Cardiovascular and Interventional Radiological Society of Europe (CIRSE) adverse event classification systems (Tables 1 and 2).^23,24^

**Table 1.** Society of Interventional Radiology’s Adverse Event Classification.^23^

Mild	No therapy or nominal therapy (eg, post-procedural imaging performed and fails to show manifestation of adverse event); near miss (eg, wrong site of patient prepared, recognized and corrected before procedure, wrong patient information entered for procedure)
Moderate	Moderate escalation of care, requiring substantial treatment under conscious sedation, blood product administration, extremely prolonged outpatient observation, or overnight admission after outpatient procedure not typical for the procedure (excludes admission or hospital days unrelated to adverse event)
Severe	Marked escalation of care, such as hospital admission or prolongation of existing hospital admission for >24 hours, hospital admission that is atypical for the procedure, inpatient transfer from regular floor/telemetry to intensive care unit, or complex intervention performed requiring general anesthesia in previously non-intubated patient (generally excludes pediatrics or in circumstances in which general anesthesia would primarily be used in lieu of conscious sedation, eg, in mentally challenged or severely uncooperative patients)
Life-threatening or disabling	Such as cardiopulmonary arrest, shock, organ failure, unanticipated dialysis, paralysis, or loss of limb or organ
Death	Patient death or unexpected pregnancy abortion

**Table 2.** Cardiovascular and Interventional Radiological Society of Europe Adverse Event Classification.^24^

Grade 1	Complication during the procedure which could be solved within the same session; no additional therapy, no post-procedure sequelae, no deviation from the normal post-therapeutic course
Grade 2	Prolonged observation including overnight stay (as a deviation from the normal post-therapeutic course <48 hours); no additional post-procedure therapy, no post-procedure sequelae
Grade 3	Additional post-procedure therapy or prolonged hospital stay (>48 hours) required; no post-procedure sequelae
Grade 4	Complication causing a permanent mild sequelae (resuming work and independent living)
Grade 5	Complication causing a permanent severe sequelae (requiring ongoing assistance in daily life)
Grade 6	Death

## Results

There were a total of 715 reported safety events. These involved Angio-Seal VIP (666; 93.1%), Evolution (41; 5.7%), STS Plus (8; 1.1%), with sizes 6F (447; 62.5%) and 8F (268; 37.5%). These ratios are reflective of the model’s prevalence in the market during the study period (2019-2020), where VIP was by far the most commonly used model, followed by Evolution and a minority included STS Plus. A minority of devices were returned to the manufacturer for root cause analysis (199; 27.8%).

Among reports, the most common procedures involved coronary intervention (238; 33.3%), coronary angiogram (62; 8.7%), peripheral intervention (67; 9.0%), peripheral angiogram (58; 8.1%), neurointervention (28; 3.9%), and coiling procedure (55; 7.7%). Of note, 2 of the entries involved brachial artery closure (off-label use for this device), whereas the remainder involved the common femoral artery.

The mode of failure involved damaged device, defined by unrecognized use of a damaged device (310; 43.4%), failed deployment (144; 20.1%), failed arterial advancement (45; 6.3%), detachment of device component during use (35; 4.9%), failed retraction (26; 3.6%), operator error (8; 1.1%), and the remainder were indeterminate (147; 20.6%) (Table 3).

**Table 3.** Mode of Device Failure Reported in the MAUDE Database Related to Angio-Seal.

Mode of failure	N (%)
Damaged device	310 (43.4)
Failed deployment	144 (20.1)
Failed arterial advancement	45 (6.3)
Detachment of device component	35 (4.9)
Failed retraction	26 (3.6)
Operator error	8 (1.1)
Indeterminate	147 (20.6)
Total	715

Percentages represent proportion of total number of included reports.

Abbreviation: MAUDE, Manufacturer and User Facility Device Experience.

The most common damaged device incidents involved (1) bypass tube found detached from the carrier tip (87 cases), (2) deformed tip of the Angio-Seal (54), (3) resistance when advancing the locator into the sheath (54), (4) missing Angio-Seal component (16), and (5) bent insertion sheath (12). Among the failed retraction category, most commonly (1) the hemostatic component was found protruding at the skin (37), (2) the collagen plug was found in the extravascular space (4), or (3) embolized intravascularly (6).

Of the 715 device complications, a total of 320 events (44.8%) were associated with patient harm (Table 4). Complications involved minor blood loss defined as <500 cc (244; 34.1%), hematoma (40; 5.6%), significant blood loss defined as >500 cc (10; 1.4%), pseudoaneurysm (10; 1.4%), thrombus (6; 0.8%), pulseless extremity (4; 0.6%), vascular dissection (3; 0.4%), emboli (2; 0.3%), and infection (1; 0.1%).

**Table 4.** Patient-Related Complications Reported in the MAUDE Database Related to Angio-Seal Use.

Complication	N (%)
Minor blood loss	244 (34.1)
Hematoma	40 (5.6)
Significant blood loss	10 (1.4)
Pseudoaneurysm	10 (1.4)
Thrombus	6 (0.8)
Pulseless extremity	4 (0.6)
Vascular dissection	3 (0.4)
Emboli	2 (0.3)
Infection	1 (0.1)
Total	320

Percentages represent proportion of total number of included reports.

Abbreviation: MAUDE, Manufacturer and User Facility Device Experience.

Manual compression was used in 43.3% of cases (310) to achieve hemostasis upon Angio-Seal failure. More advanced intervention was required in 8.8% of cases (63) to manage associated complications (Table 5). Interventions included surgical repair (31; 49.2%), thrombin injection (6; 9.5%), balloon tamponade (4; 6.3%), covered stent (3; 4.8%), and the remainder were unspecified (19; 30.2%).

**Table 5.** Interventions to Manage Complications Associated With Angio-Seal Use Reported in the MAUDE Database.

Intervention	N (%)
Surgical repair	31 (49.2)
Thrombin injection	6 (9.5)
Balloon tamponade	4 (6.3)
Covered stent	3 (4.8)
Unspecified	19 (30.2)
Total	63

Percentages represent proportion of total number of cases requiring intervention.

Abbreviation: MAUDE, Manufacturer and User Facility Device Experience.

According to SIR adverse event classification, the majority of events were associated with mild patient harm (658; 92%), followed by moderate (22; 3.1%), severe (15; 2.1%), life-threatening (18; 2.5%), and death (2; 0.3%). According to the CIRSE adverse event classification, the majority of safety events were associated with class 1 (658; 92.9%), followed by class 2 (22; 3.1%), class 3 (33; 4.6%), and class 6 (2; 0.3%) level of patient impact (Table 6). Case description of events which involve patient death or life-threatening outcomes are summarized in Table 7.

**Table 6.** Safety Event Associated Level of Patient Impact as Per the CIRSE and SIR Classifications.

SIR	N (%)	CIRSE	N (%)
Mild	658 (92.0)	Class 1	658 (92.0)
Moderate	22 (3.1)	Class 2	22 (3.1)
Severe	15 (2.1)	Class 3	33 (4.6)
Life-threatening or disabling	18 (2.5)	Class 4	0
Death	2 (0.3)	Class 5	0
		Class 6	2 (0.3)

Percentages represent proportion of total number of included reports.

Abbreviations: CIRSE, Cardiovascular and Interventional Radiological Society of Europe; SIR, Society of Interventional Radiology.

**Table 7.** Description of Cases Which Involved Patient Death or Life-Threatening/Disabling Events.

Death: Case involved a coiling neurointerventional procedure. On post-operative day 1, CT revealed a large retroperitoneal hematoma. Patient was transferred to vascular surgery service and later died. No information was given about whether this was caused by an Angio-Seal error. No issues were noted at the puncture site or with Angio-Seal use
Death: Following an unspecified procedure, a patient experienced a large retroperitoneal bleed, was taken to the operating room (OR), and later died. It was found that the internal epigastric artery was punctured at 3 spots. No information was given about whether this was caused by an Angio-Seal error
Life-threatening or disabling: A hematoma developed 12 hours post-procedure at the site of Angio-Seal use. Patient was taken to the OR to evacuate the hematoma, and collagen anchor was found in the subcutaneous fat
Life-threatening or disabling: Angio-Seal reported to have failed, and manual pressure was used to achieve hemostasis. Patient subsequently ambulated and experienced severe leg pain; no pedal pulse was appreciated. Vascular surgery was consulted, and patient was taken to OR where intravascular collagen plug was successfully removed
Life-threatening or disabling: Collagen anchor was stuck intravascularly, and the entire device could not be removed. Patient was taken to the OR for surgical removal
Life-threatening or disabling: Angio-Seal failed to achieve hemostasis and MC was subsequently applied. Ultrasound showed foreign body at the access site. Collagen anchor and suture were retrieved from the vessel during surgical removal
Life-threatening or disabling: Angio-Seal failed to achieve hemostasis and MC was subsequently applied. Angiogram study revealed foreign body at access site. Collagen anchor and suture were retrieved from the superficial femoral artery during surgery
Life-threatening or disabling: 60 hours post-cardiac catheterization, patient developed a right femoral hematoma and subsequently became hypotensive. A covered stent was used to seal the hematoma with good outcome
Life-threatening or disabling: Patient had a flow diverter treatment for an aneurysm. Upon patient reposition during the post-operative period, bleeding occurred from the site of the Angio-Seal puncture, followed by hemorrhagic shock. Manual compression was used to establish hemostasis to good effect
Life-threatening or disabling: On post-operative day 10, it was discovered that Angio-Seal anchor had embolized and occluded the vessel. The anchor was subsequently removed surgically and resulted in extended hospital stay
Life-threatening or disabling: After proper use of Angio-Seal without any issues, the collagen component intruded the blood vessel causing thrombus formation which led to vascular occlusion. Thrombus was successfully removed surgically
Life-threatening or disabling: On post-operative day 1, patient developed a pseudoaneurysm which was treated surgically
Life-threatening or disabling: Angio-Seal was used without issues. Post-procedure, patient presented to the emergency room with a retroperitoneal bleed. It was found that the anchor was in the inguinal ligament. It is unclear how this was treated
Life-threatening or disabling: 20 hours post-procedure, patient developed a hematoma at the puncture site. Upon investigation, the collagen and suture were found in the extravascular space. It is unclear how this was treated
Life-threatening or disabling: Post-cardiac catheterization, the patient’s leg was found to be pulseless and pale, requiring vascular surgery intervention. No additional information regarding cause and patient outcome was provided
Life-threatening or disabling: Angio-Seal was used without any issues. Patient developed puncture site hematoma, required blood transfusion and covered stent to treat
Life-threatening or disabling: 14 hours post-Angio-Seal deployment, patient developed retroperitoneal bleed and required surgical intervention
Life-threatening or disabling: The suture was stuck, and Angio-Seal was unable to deploy. Examination of the device showed a knot in the suture. The patient required blood transfusion
Life-threatening or disabling: 3 hours post-procedure, a hematoma developed at the puncture site. Surgical intervention was required. Anchor and collagen were found in the subcutaneous tissue of the patient

Abbreviation: CT, computed tomography; MC, manual compression.

## Discussion

The literature has shown that although complications of Angio-Seal are extremely rare, they do occur and it is important for clinicians to be aware of them.^1^ These include failure of device deployment, access-site hematoma, infected hematoma, pseudoaneurysm, arterial laceration, oozing of blood up to 1 hour post-deployment, and new onset pain at the groin.^9,25–30^ In addition, vascular occlusion, loss of distal pulse, retroperitoneal bleed, and dissection have all been described, although they remain exceedingly rare.^28–30^

The most frequent complications in our study involved minor blood loss as defined by <500 cc (34.1%), puncture site hematoma (5.6%), and significant blood loss as defined by >500 cc (1.4%). This is similar to findings of a large multicenter trial by Wong et al^9^ in which common complications were access-site rebleeding or hematoma formation; however, they had no reports of major access-site–related adverse events. The most common cause of safety events in our data was the unrecognized use of a damaged device (43.4%), which points to the simple yet crucial step of device inspection prior to use. Notably, it is possible that some cases of operator error led to a damaged device following use, which was identified and labeled retrospectively as an unrecognized use of a damaged device. The vast majority of events in our study were associated with minor adverse patient effect (92%, grade 1 level of patient impact as per the CIRSE adverse event classification). In addition, a minority of devices were returned to the manufacturer for root cause analysis (27.8%). Similarly, Khalid and Ahmad^31^ found that across a wide review of the MAUDE database, only a minority of devices were actually returned to the manufacturer. As a result, the input of the device manufacturer is lacking in many reports and a root cause analysis could not be conducted. Hence, return of device to manufacturer should be encouraged.

The majority of the complications identified in our study were similar to the prior literature. However, our study revealed some additional rare complications. These included a case of collagen caught intravascularly such that the device could not be removed, requiring surgical removal. Another case showed failure to deploy Angio-Seal due to a knot in the device suture. Although no deaths were reported in the literature to our knowledge, our data revealed 2 patient deaths reported within the Angio-Seal MAUDE data. Further analysis of these 2 cases involved retroperitoneal bleeding as a cause; however, although both reports described injury to the inferior epigastric artery found at the time of attempted rescue surgery, it remains unclear if this was related to the initial access, arterial puncture failure, or secondary to anticoagulation status. If in fact there was an inferior epigastric injury, it seems unlikely this would be related to Angio-Seal deployment or failure.

Intra-arterial dislocation of vascular plug causing arterial occlusion has been described in a number of studies.^9,25,32^ Atherosclerosis of the femoral vessels has been shown to contribute to vessel occlusion by entrapping the Angio-Seal plug in the stenotic area.^32^ Some authors suggest that Angio-Seal use should be contraindicated in cases of heavy atherosclerotic disease or vessel diameters less than 5 mm.^32^ The polymer anchor has also been reported to embolize and cause complete occlusion or stenosis of the femoral artery, requiring surgical retrieval and definitive arterial repair.^33,34^ According to the manufacturer, the product anchor is meant to be 95% absorbed by 42 days and 100% by 60 to 90 days. However, vascular obstruction has been reported up to 3 months after placement.^34^

Delayed complications of Angio-Seal are reported in the MAUDE database. For example, on post-operative day 10, the Angio-Seal anchor was found to have embolized and caused vascular occlusion, requiring surgical retrieval. In another instance, a patient presented following discharge with a retroperitoneal bleed and at the time of surgical intervention, the Angio-Seal anchor was found dislodged in the inguinal ligament. Another case involved a patient who developed a femoral hematoma and hypotension 60 hours post-cardiac intervention. A covered stent was used to seal the hematoma, although no clear cause for the hematoma was identified. Notably, the author’s experience has also seen cases of delayed hematoma whereby the anchor was found dislodged from the artery several days following device deployment. A limitation to consider is the lack of data regarding the puncture zone (vessel calcification, diameter, tortuosity) as well as puncture type (fluoroscopic or ultrasound guided vs no image guidance), which inherently affects the rates of delayed complications.

Late vascular complications of Angio-Seal have also been reported in the literature as case reports. For example, in a patient with persistent pain at access site 3 weeks following intervention, intraluminal collagen causing eccentric stenosis was found.^34^ In another case, patient presented 3 weeks post-procedure with leg claudication and was found to have intra-arterial collagen.^34^ Follow-up Dopplers did not show any absorption after 2 weeks, at which point percutaneous extraction of the collagen plug was performed, although incompletely as part of the collagen had begun to dissolve.^34^ In a similar case, angiogram 3 months post-intervention showed persistent subtotal arterial occlusion secondary to intraluminal collagen plug, subsequently treated with atherectomy and balloon angioplasty.^34^

Potential mechanisms of Angio-Seal–related arterial occlusion include the following: (1) anchor-induced dissection flap with a superimposed thrombus from dragging of the anchor along the inner arterial wall during deployment; (2) thrombosis induced by the intraluminal anchor or a portion of the collagen plug that prolapses into the lumen perhaps secondary to dynamic flexion forces; (3) atherosclerotic plaque rupture from device-induced trauma; (4) delayed detachment of the footplate from the collagen plug and its subsequent embolization; and (5) intimal hyperplasia at the closure site as a delayed cause of stenosis. Taken together, the data suggest that in patients with symptoms of arterial insufficiency following the use of Angio-Seal in both the acute and delayed stages, local arterial stenosis at the closure site should be considered in the differential diagnosis.

Moreover, risk of infection has been reported with Angio-Seal in a number of case reports whereas the overall incidence remains extremely rare.^35–37^ In theory, the collagen plug is foreign material inside the arterial lumen and can act as a nidus for infection. Proposed risk factors associated with infection are obesity, diabetes mellitus, and previous placement of percutaneous closure devices.^36^ To this end, patients with high probability of needing further vascular access at the same site in the near future may not be ideal candidates for closure device use.^36^ Angio-Seal recommendations are that vessels closed with this product not be accessed again for 6 weeks.^36^

Although it is “off-label,” the use of Angio-Seal closure for brachial access has been studied with a reported success rate of 96.9% among 140 patients.^38^ Major complications (3.1%) at 30 days consisted of puncture site hematoma, brachial artery occlusion, and pseudoaneurysm.^38^ Minor complications included minimal blood loss from the access site and mild pain at the cubital fossa.^38^ In another smaller study which included 36 patients, only minor complications including mild paresthesia, pain, and hematoma were observed.^39^

Reflecting on the data presented herein, several complications unique to Angio-Seal along with management options are discussed as follows:

1. Importance of device inspection: This analysis has shown that damaged devices account for nearly half of reported failures. Inspection of the device prior to use is therefore a simple yet crucial step in mitigating potential adverse events.
2. Collagen deposition into the artery: If this condition is suspected, the diagnosis can be confirmed by duplex ultrasound. Management is controversial; however, options include leaving the collagen plug in situ with anticoagulation, endovascular snaring, or surgical removal.^40^ In addition, intraluminal collagen deposition may remain undetected with ischemic symptoms developing several weeks after use.^41^ Therefore, all cases of lower limb ischemia within several months after Angio-Seal deployment should be investigated with duplex ultrasound to exclude intravascular collagen deposition as the potential cause.
3. Ultrasound-guided deployment: Ultrasound-guided Angio-Seal deployment has been described to be useful in 3 scenarios.^30,42^ It is useful to avoid entrapment of the anchor within posterior wall plaque or a small caliber vessel leading to intraluminal deployment of the collagen plug, to avoid extraluminal deployment in patients in whom pseudoaneurysms develop during the intervention, and to determine whether there is intraluminal deployment of the anchor and collagen plug when there is primary failure in achieving hemostasis.^42^
4. Very thin patients: Collagen may protrude from the skin after compaction has been completed. This is normal occurrence in thin patients and should not be mistaken for erroneous closure, as was the case in a number of adverse event reports in the database. If this occurs, attempt can be made to push the collagen under the skin using the compaction tube or a sterile hemostat. Alternatively, pre-puncture dissection of the subcutaneous space can be done to free the skin edges which can be sutured subsequently over the exposed plug to avoid skin protrusion. Vigorous compaction should be avoided as it may result in anchor fracture. Cutting off the excess collagen should also be avoided, as the suture woven through the collagen may be cut and the integrity of the anchor/collagen could be compromised.

### Limitations

General limitations of the MAUDE database are inclusion and selection bias given that it is a voluntary reporting system with contributions from manufactures, users, health care professionals and patients, incomplete and non-standardized reporting of details surrounding safety event, and lack of a rigorous data verification process. Moreover, although the database establishes an associative relationship between the adverse event and subsequent complication, a definitive cause and effect cannot be confirmed. In addition, a minority of devices were returned to the manufacturer, hindering root cause analysis. A degree of bias is also inherent to the process of a manufacturer-led device root cause analysis which should be considered. Furthermore, true incidence rates of safety events are unknown given the unspecified number of Angio-Seal devices used per year. Finally, the database does not reflect long-term complications.

## Conclusions

We present safety events encountered in real-world practice with Angio-Seal. The MAUDE database captures real-world device malfunctions not typically appreciated in conventional clinical trials. The database provides valuable feedback for manufacturers to optimize product design and direct manufacturer-user training. Moreover, the data provide valuable insight for clinician-users on managing common device malfunctions to foster patient safety. Ideally, greater detail would be available in safety reports along with return of device to manufacturer in order to facilitate in-depth root cause analysis of safety events. System-level strategies for addressing barriers to under-reporting of safety events include ensuring that clinicians are aware of the processes involved in reporting and simplifying the process. From a policy perspective, aligning legislation to foster a system that mandates reporting of adverse events is central to making MAUDE and similar databases more impactful. Equally important is the reporting of near misses as awareness of the factors that might lead to error is the first key to error reduction.^43^

## References

[1] Noori VJ Eldrup-Jørgensen J. A systematic review of vascular closure devices for femoral artery puncture sites. J Vasc Surg. 2018;68(3):887–899. doi:10.1016/j.jvs.2018.05.019. 30146036

[2] Proficient Market Insights. Vascular closure devices market size 2022 Growth projection of (USD 1,735.13 million) evident for upcoming opportunities in the industry with massive ROI for investors. GlobeNewswire. https://www.globenewswire.com/news-release. Published April 7, 2022. Accessed July 10, 2022.

[3] Sarin SN Shah RK Chun A , et al. Use of the 8-F Angio-Seal vascular closure device in large-caliber arteriotomies. J Endovasc Ther. 2012;19(4):497–500. doi:10.1583/12-3881.1. 22891829

[4] Veasey RA Large JK Silberbauer J , et al. A randomised controlled trial comparing StarClose and AngioSeal vascular closure devices in a district general hospital—the SCOAST study. Int J Clin Pract. 2008;62(6):912–918. doi:10.1111/j.1742-1241.2008.01761.x. 18479284

[5] Applegate RJ Turi Z Sachdev N , et al. The Angio-Seal evolution registry: outcomes of a novel automated Angio-Seal vascular closure device. J Invasive Cardiol. 2010;22(9):420–426. 20814049

[6] Hvelplund A Jeger R Osterwalder R , et al. The Angio-Seal™ femoral closure device allows immediate ambulation after coronary angiography and percutaneous coronary intervention. EuroIntervention. 2011;7(2):234–241. doi:10.4244/EIJV7I2A38. 21646066

[7] Ketterle J Rittger H Helmig I , et al. Comparison of Exo-Seal(®) and Angio-Seal (®) for arterial puncture site closure: a randomized, multicenter, single-blind trial. Herz. 2015;40(5):809–816. doi:10.1007/s00059-015-4306-3. 26070467

[8] Rastan A Sixt S Schwarzwälder U , et al. VIPER-2: a prospective, randomized single-center comparison of 2 different closure devices with a hemostatic wound dressing for closure of femoral artery access sites. J Endovasc Ther. 2008;15(1):83–90. doi:10.1583/07-2253.1. 18254672

[9] Wong HF Lee CW Chen YL , et al. Prospective comparison of Angio-Seal versus manual compression for hemostasis after neurointerventional procedures under systemic heparinization. AJNR Am J Neuroradiol. 2013;34(2):397–401. doi:10.3174/ajnr.A3226. 22859279 PMC7965118

[10] Johnson DT Durack JC Fidelman N , et al. Distribution of reported StarClose SE vascular closure device complications in the manufacturer and user facility device experience database. J Vasc Interv Radiol. 2013;24(7):1051–1056. doi:10.1016/j.jvir.2013.03.032. 23796092

[11] Villalba L Witter J. Rofecoxib, Merck, and the FDA. N Engl J Med. 2004;351(27):2875–2878. 15625745

[12] Pentecost MJ. Vioxx, radiology, and the food and drug administration. J Am Coll Radiol. 2005;2(5):394–397. doi:10.1016/j.jacr.2005.01.019. 17411841

[13] Gurtcheff SE. Introduction to the MAUDE database. Clin Obstet Gynecol. 2008;51(1):120–123. doi:10.1097/GRF.0b013e318161e657. 18303506

[14] US Food & Drug Administration. MAUDE—manufacturer and user facility device experience. Date unknown. https://www.accessdata.fda.gov/scripts/cdrh/cfdocs/cfmaude/search.cfm.

[15] Case BC Forrestal BJ Yerasi C , et al. Real-world experience of the sentinel cerebral protection device: insights from the FDA manufacturer and user facility device experience (MAUDE) database. Cardiovasc Revasc Med. 2020;21(2):235–238. doi:10.1016/j.carrev.2019.11.014. 31780421

[16] Khalid N Javed H Ahmad SA , et al. Analysis of the food and drug administration manufacturer and user facility device experience database for patient- and circuit-related adverse events involving extracorporeal membrane oxygenation. Cardiovasc Revasc Med. 2020;21(2):230–234. doi:10.1016/j.carrev.2019.11.011. 31767523

[17] Shlofmitz E Garcia-Garcia HM Rogers T , et al. Techniques to optimize the use of optical coherence tomography: insights from the manufacturer and user facility device experience (MAUDE) database. Cardiovasc Revasc Med. 2019;20(6):507–512. doi:10.1016/j.carrev.2019.03.009. 30962083

[18] Khalid N Javed H Rogers T , et al. Adverse events with orbital atherectomy: an analytic review of the MAUDE database. Eurointervention. 2020;16(4):e325–e327. doi:10.4244/EIJ-D-19-00295. 31422928

[19] Chen Y Shah AA Shlofmitz E , et al. Adverse events associated with the use of guide extension catheters during percutaneous coronary intervention: reports from the manufacturer and user facility device experience (MAUDE) database. Cardiovasc Revasc Med. 2019;20(5):409–412. doi:10.1016/j.carrev.2019.02.016. 31079818

[20] Khalid N Javed H Rogers T , et al. Adverse events and modes of failure related to the FilterWire EZ Embolic Protection System: lessons learned from an analytic review of the FDA MAUDE database. Catheter Cardiovasc Interv. 2019;94(1):157–164. doi:10.1002/ccd.28297. 30985082

[21] Khalid N Rogers T Shlofmitz E , et al. Adverse events and modes of failure related to Impella RP: insights from the manufacturer and user facility device experience (MAUDE) database. Cardiovasc Revasc Med. 2019;20(6):503–506. doi:10.1016/j.carrev.2019.03.010. 30922871

[22] Khalid N Rogers T Shlofmitz E , et al. Adverse events and modes of failure related to the Impella percutaneous left ventricular assist devices: a retrospective analysis of the MAUDE database. Eurointervention. 2019;15(1):44–46. doi:10.4244/EIJ-D-18-01021. 30803939

[23] Khalilzadeh O Baerlocher MO Shyn PB , et al. Proposal of a new adverse event classification by the Society of Interventional Radiology Standards of Practice Committee. J Vasc Interv Radiol. 2017;28(10):1432–1437.e3. doi:10.1016/j.jvir.2017.06.019. 28757285

[24] Filippiadis DK Binkert C Pellerin O , et al. CIRSE quality assurance document and standards for classification of complications: the CIRSE classification system. Cardiovasc Intervent Radiol. 2017;40(8):1141–1146. doi:10.1007/s00270-017-1703-4. 28584945

[25] Upponi SS Ganeshan AG Warakaulle DR , et al. Angioseal versus manual compression for haemostasis following peripheral vascular diagnostic and interventional procedures—a randomized controlled trial. Eur J Radiol. 2007;61(2):332–334. doi:10.1016/j.ejrad.2006.09.007. 17071040

[26] Hermanides RS Ottervanger JP Dambrink JH , et al. Closure device or manual compression in patients undergoing percutaneous coronary intervention: a randomized comparison. J Invasive Cardiol. 2010;22(12):562–566. 21127358

[27] Nikolsky E Mehran R Halkin A , et al. Vascular complications associated with arteriotomy closure devices in patients undergoing percutaneous coronary procedures: a meta-analysis. J Am Coll Cardiol. 2004;44(6):1200–1209. doi:10.1016/j.jacc.2004.06.048. 15364320

[28] Jud P Portugaller R Bohlsen D , et al. Successful retrieval of an embolized vascular closure device (Angio-Seal®) after peripheral angioplasty. Cardiovasc Intervent Radiol. 2017;40(6):942–946. doi:10.1007/s00270-017-1565-9. 28101616 PMC5409923

[29] Gregory D Midodzi W Pearce N. Complications with Angio-Seal™ vascular closure devices compared with manual compression after diagnostic cardiac catheterization and percutaneous coronary intervention. J Interv Cardiol. 2013;26(6):630–638. doi:10.1111/joic.12070. 24125119

[30] Kalapatapu VR Ali AT Masroor F , et al. Techniques for managing complications of arterial closure devices. Vasc Endovascular Surg. 2006;40(5):399–408. doi:10.1177/1538574406293760. 17038574

[31] Khalid N Ahmad SA. Use and application of MAUDE in patient safety. In: Aboubakr S (ed) StatPearls. Treasure Island, FL: StatPearls Publishing; 2022, pp. 1–4. 34033344

[32] Kadner A Schmidli J Schwegler I , et al. Complications associated with the arterial puncture closure device—Angio-Seal. Vasc Endovascular Surg. 2008;42(3):225–227. doi:10.1177/1538574407312657. 18230871

[33] Eidt JF Habibipour S Saucedo JF , et al. Surgical complications from hemostatic puncture closure devices. Am J Surg. 1999;178(6):511–516. doi:10.1016/s0002-9610(99)00246-9. 10670863

[34] Goyen M Manz S Kröger K , et al. Interventional therapy of vascular complications caused by the hemostatic puncture closure device angio-seal. Catheter Cardiovasc Interv. 2000;49(2):142–147. doi:10.1002/(sici)1522-726x(200002)49:2<142::aid-ccd5>3.0.co;2-g. 10642760 10.1002/(sici)1522-726x(200002)49:2<142::aid-ccd5>3.0.co;2-g

[35] Whitton Hollis H Jr Rehring TF. Femoral endarteritis associated with percutaneous suture closure: new technology, challenging complications. J Vasc Surg. 2003;38(1):83–87. doi:10.1016/s0741-5214(03)00126-5. 12844094

[36] Franco J Motaganahalli R Habeeb M , et al. Risk factors for infectious complications with angio-seal percutaneous vascular closure devices. Vascular. 2009;17(4):218–221. doi:10.2310/6670.2009.00017. 19698303

[37] Cooper CL Miller A. Infectious complications related to the use of the angio-seal hemostatic puncture closure device. Catheter Cardiovasc Interv. 1999;48(3):301–303. doi:10.1002/(sici)1522-726x(199911)48:3<301::aid-ccd15>3.0.co;2-f. 10525234 10.1002/(sici)1522-726x(199911)48:3<301::aid-ccd15>3.0.co;2-f

[38] Lupattelli T Clerissi J Clerici G , et al. The efficacy and safety of closure of brachial access using the AngioSeal closure device: experience with 161 interventions in diabetic patients with critical limb ischemia [published correction appears in J Vasc Surg. 2008;48(3):778]. J Vasc Surg. 2008;47(4):782–788. doi:10.1016/j.jvs.2007.11.050. 18295438

[39] Bilecen D Bongartz G Ostheim-Dzerowycz W. Off-label use of Angio-Seal vascular closure device for brachial artery puncture closure-deployment modification and initial results after transbrachial PTA. Eur J Vasc Endovasc Surg. 2006;31(4):431–433. doi:10.1016/j.ejvs.2005.05.030. 16442818

[40] Stein BC Teirstein PS. Nonsurgical removal of angio-seal device after intra-arterial deposition of collagen plug. Catheter Cardiovasc Interv. 2000;50(3):340–342. doi:10.1002/1522-726x(200007)50:3<340::aid-ccd15>3.0.co;2-h. 10878634 10.1002/1522-726x(200007)50:3<340::aid-ccd15>3.0.co;2-h

[41] Dregelid E Jensen G Daryapeyma A. Complications associated with the Angio-Seal arterial puncture closing device: intra-arterial deployment and occlusion by dissected plaque. J Vasc Surg. 2006;44(6):1357–1359. doi:10.1016/j.jvs.2006.07.034. 17145443

[42] Ravi R Chan TY Shaikh UH , et al. Ultrasound-guided angio-seal deployment. J Vasc Interv Radiol. 2015;26(3):444–446. doi:10.1016/j.jvir.2014.10.011. 25735527

[43] Mafeld S Musing ELS Conway A , et al. Avoiding and managing error in interventional radiology practice: tips and tools. Can Assoc Radiol J. 2020;71(4):528–535. doi:10.1177/0846537119899215. 32100547

